# A Case of Autosomal Recessive Intellectual Developmental Disorder Type 5 Presenting with Epilepsy

**DOI:** 10.1155/2022/4056780

**Published:** 2022-11-14

**Authors:** Mahpara Hasan, Gayatra Mainali, Ermal Aliu, Sita Paudel

**Affiliations:** ^1^Pennsylvania State Health College of Medicine, Hershey, PA 17033, USA; ^2^Department of Pediatrics, Division of Neurology, Pennsylvania State Health College of Medicine, Hershey, PA 17033, USA; ^3^Department of Pediatrics, Pennsylvania State Health College of Medicine, Hershey, PA 17033, USA

## Abstract

Autosomal recessive intellectual developmental disorder type 5 (MRT5, OMIM # 611091) is caused by biallelic pathogenic variants, leading to loss of function of the NSUN2 gene which encodes a methyltransferase involved in several biological processes, ranging from stress response to neurodevelopment (Hussain 2021). The current literature shows that MRT5 typically manifests with intellectual disability, facial dysmorphism, juvenile cataracts, chronic nephritis, hearing impairment, seizures, cerebellar atrophy, and microcephaly (Pingree et al. 2021). We describe a case of a patient with MRT5 who developed epilepsy in his teens, a rare clinical presentation that has not yet been discussed at length in the literature. Our patient is a 15-year-old male with a history of autism, developmental delay, and focal epilepsy who underwent genetic testing and was found to have a homozygous frameshift mutation in NSUN2 predicted to cause loss of function. This case emphasizes that epilepsy can be a phenotypic manifestation in patients with MRT5.

## 1. Introduction

NOP2/Sun RNA Methyltransferase 2 (NSUN2) is a gene located on the short arm of chromosome 5 and encodes a methyltransferase that is required for splicing at C34 of tRNA leu (CAA) and C47 and C48 of tRNA-asp (GTC) [2]. This allows for the stabilization of the anticodon-codon pairing and allows for the correct translation of mRNA [3]. Mutations in the NSUN2 gene have led to a range of clinical presentations including intellectual disability, facial dysmorphisms, juvenile cataracts, chronic nephritis, hearing impairments, seizures, cerebellar atrophy, and microcephaly [2, 4, 5].

A proposed mechanism for the link between MRT5 and neurodevelopmental disorders postulates that the loss of 5-methylcytosine increases the angiogenin-mediated endonucleolytic cleavage of tRNAs, leading to an accumulation of 5′ tRNA-derived small RNA fragments. Accumulation of these fragments reduces the rate of protein translation and can activate stress pathways that lead to reduced cell size and increased apoptosis of cortical, hippocampal, and striatal neurons [1].

While the relationship between MRT5 and intellectual disability has been well established, there is limited information describing the association between NSUN2 and epilepsy. Martinez et al. identified an NSUN2 variant in the homozygous state, resulting in a Dubowitz-like syndrome characterized by mild microcephaly, intellectual disability, and peculiar facies in three affected family members of Lebanese origin. Of the three affected individuals, only the eldest displayed epilepsy, and seizures were controlled with Valproic acid [4].

Since the current literature has not detailed the association between MRT5 and epilepsy in-depth, the goal of this case report is to describe the relationship between MRT5 and epilepsy as a phenotypic manifestation, albeit a rare one.

## 2. Case

This patient was born full-term from nonconsanguineous Nepalese parents in Nepal. The mother was a gravida 1 para 1 (G1P1) female with an unremarkable pregnancy. The patient's postnatal course was unremarkable. Developmental delays were first appreciated around 1 year of age. He sat up on his own at 12 months, started combat crawling at 2 years, and walked at 3 years with an ataxic gait. He moved to the USA at 5 years of age. He has never been verbal and his intelligence quotient (IQ) was found to be approximately 40. Based on these findings, he was diagnosed with significant intellectual disability and autism. He was evaluated by an outside geneticist at 8 years of age with concern for dysmorphic features, significant microcephaly, and sensorineural and conductive hearing loss. On exam, inverted nipples and shawl scrotum were observed. He underwent a genetic workup including karyotype, microarray, fragile *X* testing, methylation study for Angelman syndrome, and biochemical labs which were nondiagnostic.

At 11 years of age, he underwent an electroencephalogram (EEG) at a different facility due to concern about night-time agitation and staring episodes. Reportedly EEG was abnormal and showed diffuse slowing along with focal spike and slow wave discharges without any electrographic seizures. Antiseizure medications were not started.

**Figure 1:**
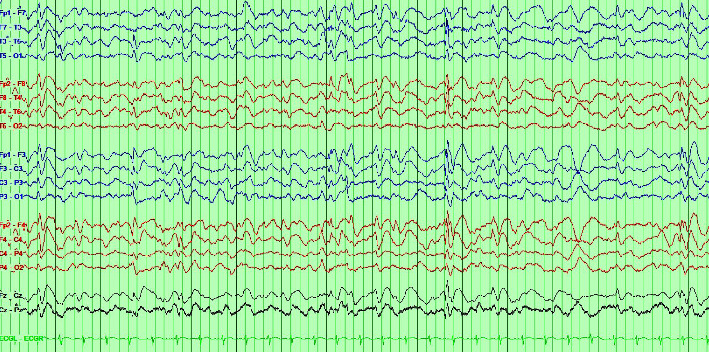
Interictal EEG shows diffuse slowing along with bursts of generalized spike and slow wave discharges.

At 14 years of age, he presented with a new type of seizure characterized by impaired consciousness, drooling, eye deviation, and mouth twitching. The left side of his body would also hunch over. Each episode would last for 15–20 minutes and would often be followed by sleepiness. These episodes occurred almost on a daily basis. He underwent 24 hours ambulatory EEG and interictal findings showed frequent generalized 1–2.5 Hz spike and slow wave discharges (Figure 1) and sleep-activated focal epileptiform discharges without any electrographic seizures. He was started on Valproic acid. The dose was slowly titrated up to 750 mg twice daily (30 mg/kg/day) but his seizures remained uncontrolled. As a result, Clobazam was added after 6 months. Currently, he is on Clobazam 10 mg twice daily along with Valproic acid and this has been effective so far.

Work-up was initiated to evaluate the etiology of the seizures. Brain MRI was unremarkable. Genetic testing, using an Autism/ID Xpanded panel (GeneDx, Bethesda MD), identified a homozygous variant (c.560del; p.Pro187Leufs*∗*8, NM_017755.5) in the NSUN2 gene confirmed by Sanger sequencing. This frameshift variant is predicted to result in the loss of function of NSUN2. Parental studies were only carried out on his mother, which confirmed she was a carrier for the pathogenic variant. The other variant was assumed to be paternally inherited, however, this was not confirmed.

At baseline, the patient is nonverbal, cognitively delayed, and does not follow commands. He has increased tone throughout, with strength against gravity in all extremities. Deep tendon reflexes are 2+ throughout without clonus. He ambulates mainly with a wheelchair, although he can walk short distances with assistance and he has a wide-based spastic gait. He has a gastrostomy tube for feeding as he is unable to eat anything by mouth. He has not been toilet trained and he wears diapers.

## 3. Discussion

We describe a case of epilepsy due to MRT5 caused by biallelic mutations resulting in a loss of function of the NSUN2 gene which encodes a methyltransferase involved in tRNA activity and mRNA translation. Failure of NSUN2-mediated tRNA methylation leads to the accumulation of 5′tRNA-derived small RNA fragments which trigger stress pathways that lead to reduced translation, reduced cell size, and increased apoptosis of cortical, hippocampal, and striatal neurons [2]. Current literature has elucidated the relationship between MRT5 and intellectual disability, however, there is limited research investigating the association between the NSUN2 mutation and epilepsy in depth.

The case presented in this report describes a 15-year-old patient with a history of autism, developmental delay, and focal seizures. An autism/intellectual disability gene panel showed that the patient has a homozygous frameshift pathogenic variant in NSUN2, c.560del; p.Pro187Leufs*∗*8, that is predicted to result in a loss of function of the mature protein causing MRT5. There are 25 patients in the reported literature that have MRT5, the majority of which are caused by out-of-frame variants resulting in a loss of function [2]. These individuals present with facial dysmorphism, microcephaly, hypertonia, and short stature. The variant identified in this patient has not been reported before. Only one other case in the literature has documented seizures; however, this patient was homozygous for a (c.538-1G > *C*, NM_017755.6) variant which was located in an intronic region, and the nature of the epileptic seizures was not characterized extensively [4]. The case in this report highlights the importance of incorporating genetic testing such as gene-based panels and whole exome sequencing in the initial workup for cases of epilepsy with a suspected genetic basis. In addition, the seizure medication regimen that was utilized by this patient (Valproic acid and Clobazam) presents as a viable combination to control seizures related to MRT5.

## Data Availability

The authors confirm that the data supporting the findings of this study are available within the article (and/or) its supplementary materials.
